# Nurse Practitioners Navigating the Consequences of Directives, Policies, and Recommendations Related to the COVID-19 Pandemic in Long-Term Care Homes

**DOI:** 10.1177/07334648221110210

**Published:** 2022-11

**Authors:** Katherine S. McGilton, Alexandra Krassikova, Aria Wills, Vanessa Durante, Lydia Yeung, Shirin Vellani, Souraya Sidani, Astrid Escrig-Pinol

**Affiliations:** 1 KITE, 7961 Toronto Rehabilitation Institute – University Health Network, Toronto, ON, Canada; 2 Lawrence S. Bloomberg Faculty of Nursing, University of Toronto, Toronto, ON, Canada; 3 Rehabilitation Sciences Institute, Faculty of Medicine, University of Toronto, Toronto, ON, Canada; 4 Daphne Cockwell School of Nursing, 104269 Ryerson University, Toronto, ON, Canada; 5 Mar Nursing School (ESIMar), Pompeu Fabra University, Barcelona, Spain

**Keywords:** nurse practitioner, long-term care, nursing home, COVID-19, models of care

## Abstract

**Objectives:**

New models for the workforce are required in long-term care (LTC) homes, as was made evident during the Coronavirus Disease 2019 (COVID-19) pandemic. Nurse Practitioner (NP)-led models of care represent an effective solution. This study explored NPs’ roles in supporting LTC homes as changes in directives, policies, and recommendations related to COVID-19 were introduced.

**Design:**

Qualitative exploratory study.

**Context:**

Thirteen NPs working in LTC homes in Ontario, Canada.

**Methods:**

Semi-structured interviews were conducted in March/April 2021. A five-step inductive thematic analysis was applied.

**Findings:**

Analysis generated four themes: leading the COVID-19 vaccine rollout; promoting staff wellbeing related to COVID-19 fatigue; addressing complexities of new admissions; and negotiating evolving collaborative relationships.

**Conclusions:**

Nurse practitioners were instrumental in supporting LTC homes through COVID-19 regulatory changes producing unintended consequences. The NPs’ leadership in transforming care is equally essential in LTC homes as in other established healthcare settings, such as primary and acute care.

**What this paper adds**

• Nurse practitioners working in long-term care effectively responded to directive, policy, and recommendation changes during the COVID-19 pandemic by pivoting within their role and coordinating responses with the leadership team.
• Nurse practitioners continued to employ their leadership, nursing, and clinical expertise to support residents, staff, families, and administrators through the second wave of the COVID-19 pandemic.

**Applications of study findings**

• Given the findings of previous work and the findings of this study, nurse practitioners should be considered a mandatory member of the long-term care workforce.
• New models of collaborative care must be considered to optimize the nurse practitioner role within the long-term care home.

## Background

As long-term care (LTC) homes were disproportionately affected by the Coronavirus Disease 2019 (COVID-19) pandemic (Stall et al., 2021), it was critical to develop solutions addressing the challenges facing resident care in this sector. The essential role of Nurse Practitioners (NPs) has been greatly underscored during the COVID-19 pandemic as a lynchpin to quality in LTC homes (Rosa et al., 2020; Vellani et al., 2021). NPs are graduate-trained advanced practice nurses, who work in diverse healthcare settings, including LTC homes (International Council of Nurses [ICN], 2018). NPs can autonomously provide care to their patients, diagnose, create treatment plans, and prescribe medications, including controlled drugs and substances (Ontario Ministry of Health and Long-Term Care [M.o.L.T.C.], 2017). The NP role evolved throughout the pandemic with practice changes imposed by health and social directives, policies, and recommendations to contain the spread of COVID-19. In order to provide timely clinical care to the residents, in Ontario, Canada an emergency management act authorized NPs to act regularly as most responsible providers (MRPs) and to work as Medical Directors in LTC homes, a role conventionally reserved for physicians, whose in-person attendance was limited in favor of virtual care (Government of Ontario, 2019a, Ontario Ministry of Health, 2020a). The scope of practice of NPs in the United States was similarly extended through flexibility in regulation (Thomas-Gayle & Muller, 2021). NPs continued to deliver positive resident outcomes during the COVID-19 pandemic notwithstanding the expansion in their scope of practice and responsibilities (McGilton et al., 2021; Mileski et al., 2020; Vellani et al., 2021).

Underscoring their integral role, NPs stepped in to minimize the suffering of residents, moral distress of staff, and grief experienced by families. In close collaboration with LTC home staff, NPs provided comprehensive care to residents including timely advance care planning and goals of care discussions; palliative and end-of-life care; as well as facilitated virtual visits with physician specialists (Diez-Sampedro et al., 2020; McGilton et al., 2021; Vellani et al., 2021). Thus, the regulatory changes enacted during the pandemic expanded the practice authority of NPs, and testified anew to their capacity to function as exemplary independent clinicians who are effective in promoting interdisciplinary collaboration and ensuring high quality care for LTC home residents (O’Reilly-Jacob et al., 2022).

As a myriad of chronic structural and systemic issues in the LTC sector have been brought into focus throughout the COVID-19 pandemic (McGilton et al., 2020), the ongoing challenges faced by the LTC workforce in providing optimal care for residents with complex needs continued to be highlighted. In an effort to elucidate these challenges, during wave one of the pandemic we conducted interviews with NPs to describe their experience in LTC homes, and the results have been published elsewhere (McGilton et al., 2021; Vellani et al., 2021). Although the role of NPs and their positive influence on outcomes during the first wave of COVID-19 pandemic has been articulated in previous work, the NPs’ contribution during the subsequent wave was less clear. With the health and social directives, policies, and recommendations changing further in response to the evolving COVID-19 pandemic, the current study aimed to explore the roles and responsibilities of NPs during the second wave of the pandemic, declared in September 2020 by the Government of Canada. The study objective was to examine NPs’ responses and changes in practice related to evolving directives, policies, and recommendations during the second wave of the pandemic.

## Methods

### Design & Data Collection

An exploratory qualitative study was designed to assess the roles and experiences of NPs working in LTC homes (Patton, 2001). Semi-structured interviews were conducted by the Research Coordinator (AK – BSc) over the telephone with NPs between March and April 2021 to explore the changes in their role since the beginning of the second wave of the pandemic. A semi-structured interview guide was employed to prompt NPs to think about their experiences (see Supplemental Appendix 1) (Patton, 2001). Field notes were taken concurrently to the interviews, which were audio recorded and transcribed verbatim, lasting 40 minutes on average.

Recruitment of NPs was facilitated by the Nurse Practitioners Association of Ontario (NPAO). Thirteen NPs provided informed consent in writing to be re-interviewed from our previous sample, with which data saturation was reached, while one NP declined due to being on parental leave (McGilton et al., 2021). Most participants were women, working full-time, with 9 years of experience on average as an NP. The characteristics of participants and their respective LTC homes are summarized in Table 1. The study protocol was approved by the University Health Network Research Ethics Board (20-5652).

**Table 1.** Participant Characteristics.

Participant characteristics	*n* (%) or Average (Range)
Age (years)	45.5 (28–66)
Gender	
Men	3 (23%)
Women	10 (76%)
Work experience (years)	9.9 (2–12)
Specialty	
Primary health care	8 (62%)
Adult	4 (31%)
Both	1 (7%)
Employment type	
Attending nurse practitioner^a^	7 (54%)
Nurse practitioner-led outreach team^b^	6 (46%)
Site-specific characteristics	
Location of LTCH	
Rural	4 (31%)
Urban	9 (69%)
LTCH ownership	
For-profit	6 (46%)
Not-for-profit	7 (54%)

^a^Attending NP—NPs dedicated as primary care providers, working onsite in Ontario LTC homes to provide improved access, quality and continuity of care.

^b^Nurse Practitioner-led Outreach Teams—mobile teams comprised of NPs and nurses who provide comprehensive acute and episodic care to residents of Ontario LTC homes.

### Analysis

An inductive thematic analysis was applied to identify patterns emerging from the data (Braun & Clarke, 2006). Initial themes were first defined using reflexive thematic analysis (Braun & Clarke, 2006). The analysis team, composed of the RC and additional analysts (AW, VD, LY, and NZ), generated a list of four broad themes based on the topics identified by the RC and a note taker (LY) during the interviews. Each transcript was then independently coded using NVIVO by primary (AW) and secondary (VD) analysts. The entire dataset was systematically coded into the initial themes. The research team in full reviewed each theme to identify six sub-themes contained within. For instance, when participants spoke about complexities surrounding new admissions, NPs discussed the demand to manage the needs of newly admitted residents, so we included this concept in the sub-theme “managing residents’ needs caused by isolation at admission”. Decisions regarding the choice of themes were not made by quantifying the number of times a single topic was mentioned by participants. Rather, the research team assessed its relevance qualitatively, by getting acquainted with the data through transcript reading and debriefing sessions, through which the themes list was generated. In line with the methodology of Braun and Clarke (2006), the team reviewed the themes and sub-themes for coherence, reflecting NPs’ ubiquitous leadership, nursing and clinical capacities underlying each theme, and adequate and meaningful differences from each other to reflect specific changes in NPs’ roles, in order to present the story in the data. Themes and sub-themes were named and defined. In accordance with suggestions of experts in qualitative methodology (Patton, 2001), we ensured rigorous trustworthiness and credibility through holding debriefing sessions with peers; practicing reflexivity and triangulation; maintaining a detailed record of the analysis process; systematically managing data; and reviewing different accounts. We followed the Standards for Reporting Qualitative Research guidelines as detailed by O’Brien et al. (2014) throughout the study process.

## Findings

Four themes related to changes experienced by NPs to their roles during the second wave of the pandemic were identified: (a) leading the COVID-19 vaccine rollout, (b) promoting staff wellbeing related to COVID-19 fatigue, (c) addressing complexities surrounding new admissions, and (d) negotiating evolving collaborative relationships. Although the identified themes are discrete, they share common elements in employing NPs’ competencies grounded in their nursing and leadership preparation to respond to changes in LTC due to COVID-19 directives, policies, and recommendations. Specific directives, policies, and recommendations enacted during the second wave of the pandemic are presented in Table 2 alongside the four themes and their respective sub-themes.

**Table 2.** Directives, Policies, and Recommendations, Associated Themes, Sub-Themes, and Illustrative Quotations Related to Nurse Practitioners’ Role During Second Wave of the COVID-19 Pandemic.

Theme	Directive/Policy/Recommendation	Sub-Theme	Illustrative Quotation
Leading the COVID-19 vaccine roll-out	Directive: Every licensee of a long-term care home shall ensure that there is in place a written policy on COVID-19 immunization aimed at supporting education and informed choice about COVID-19 vaccination and shall ensure that the policy is complied with^a^	Vaccine administration and monitoring for side effects	“So, I’ve been pretty much the lead in preparing and ensuring the good roll out of vaccination clinics…But I’ve also been involved in just supervising the clinic as it’s happening, the day of” (NP 03)
	Policy: All staff are required (a) proof of COVID-19 vaccine administration, (b) written proof of a medical reason, provided by either a physician or registered nurse in the extended class, that sets out that the person cannot be vaccinated against COVID-19, or (c) proof that the individual has completed an educational program related to COVID-19 vaccination approved by the licensee^a^	Minimizing vaccine hesitancy	“[The LTC Home] asked me talk to staff on the issue of vaccine hesitancy. Just more or less educating them on the vaccine, and what it does and what to expect, and what are the side effects because there was actually quite a bit of hesitancy at first” (NP 12)
	Recommendation: NPs are to participate in immunization as vaccinators and recipients, counsel patients, address patient concerns and questions, and combat myths^b^	—	—
	Recommendation: Designate an individual to assess residents/staff/essential caregivers/support workers post-vaccine^c^	—	—
	Recommendation: Encourage/support COVID-19 vaccination by providing education to workers^d^	—	—
Promoting staff wellbeing during COVID-19 fatigue	Directive: Every LTC home in Ontario is legally required to have an IPAC program as part of their operations^e^	Addressing COVID-19 fatigue	“I think my being back at home has changed their use of masks…I think their mask use has improved with someone that isn’t writing them up but can comment to them about proper use. And I think that’s really what people need. Not punitive, but…continual good management” (NP 13)
	Policy: Staff and visitors must be actively screened [with rapid antigen testing] once per day at the beginning of their shift or visit. Staff, student placements and volunteers who enter LTC homes two or more days in a 7-day period undergo antigen tests on non-consecutive days up to 3 times in the period prior to entry into the LTC home. A negative antigen test result is required according to the above frequencies before individuals are permitted to enter the home and have contact with a resident or another “cleared” person^f^	Provision of emotional support	“I tend to get called in if somebody’s not having a good day anyway. And we talk and make a plan. I don’t treat anybody specifically, but we talk openly. I try to talk openly about how I’m feeling so that everybody else can” (NP 06)
	Policy: Appropriate eye protection (e.g., goggles or face shield) is required for all staff and essential visitors when providing care to residents with suspect/confirmed COVID-19 and in the provision of direct care within 2 m of residents in an outbreak area^e^	—	—
	Recommendation: Provide training to home staff, including temporary/agency staff and staff/volunteers from external partners, with respect to outbreak prevention and control measures, including IPAC measures and the use of personal protective equipment^d^	—	—
Admitting new residents	Directive: The receiving LTC home must have a plan for individuals being admitted/transferred as part of the legally mandated IPAC program^e^	Managing residents’ needs caused by isolation at admission	“[Isolation protocols have] definitely taken all the human aspect of having new residents coming in. We typically try to make them feel like they’re at home and keep them close-by and everything. But now we’re not even able to do that” (NP 03)
	Policy: Individuals must be placed in a single room on admission to complete their 14-day self-isolation under droplet and contact Precautions^e^	—	—
	Recommendation: Individuals who may have challenges with isolation due to a medical condition (e.g., dementia) are not to be denied admission or transfer on this basis alone^f^	—	—
Negotiating evolving collaborative relationships	Recommendation: While virtual care will continue to part of routine physician practice, some of the pressures that existed early in the pandemic that required prioritizing virtual care (e.g., no COVID vaccines, lack of PPE) have now diminished. With appropriate PPE, in most instances, in-person physician care can now be provided safely and appropriately^g^	Collaboration with physicians	“[W]e never have any conflicts…They’re fine with the decisions that I’ve made and grateful that somebody’s got eyes on them, on a more regular basis…We have a very good working relationship” (NP 11)
	—	—	“[Geriatric psychiatrist] is always helpful. Always responds to my calls and always there to help me out. It’s a great relationship what we have…We had it prior, but it was strengthened during the pandemic” (NP 01)

^a^Ontario Ministry of Health and Long-Term Care. (2021) *Minister’s directive: Long-term care home COVID-19 immunization policy.* Government of Ontario. https://www.ltchomes.net/LTCHPORTAL/Content/Snippets/Minister's_Directive_LTCH_COVID_vaccination_policy_2021-05-31_FINAL_2.pdf.

^b^COVID-19: Guidance for prioritizing health care workers for COVID-19 vaccination. Government of Ontario. https://www.health.gov.on.ca/en/pro/programs/publichealth/coronavirus/docs/Guidance_for_Prioritizing_HCW_covid19_vaccination_2020-01-08.pdf.

^c^Ontario Ministry of Health and Long-Term Care (2021). Initial COVID-19 immunization readiness checklist to support preparations in long-term care and retirement homes. Government of Ontario. https://ltchomes.net/LTCHPORTAL/Content/Snippets/Initial_COVID-19_Immunization_Readiness_Checklist_to_Support_Preparations_in_Long-Term_Care_and_Retirement_Homes.pdf.

^d^COVID-19 Guidance: Long-term care homes and retirement homes for public health units. Government of Ontario. https://www.health.gov.on.ca/en/pro/programs/publichealth/coronavirus/docs/2019_LTC_homes_retirement_homes_for_PHUs_guidance.pdf

^e^Ontario Ministry of Health and Long-Term Care. (2021). Enhancing the protection for ltc homes through rapid antigen testing and third party oversight memo. Government of Ontario.https://www.ltchomes.net/LTCHPORTAL/Content/Snippets/MLTC_Assoc_DM_Memo_re_Enhancing_Protection_for_LTC_Homes-v1.0-2021-01-27.pdf.

^f^Ontario Ministry of Health and Long-Term Care. (2020a)​. Directive #3 for long-term care homes under the long-term care homes act, 2007. Government of Ontario. https://www.health.gov.on.ca/en/pro/programs/publichealth/coronavirus/docs/directives/LTCH_HPPA.pdf

^g^Ontario College of Family Physicians (2021). Considerations for family physicians: Balancing in-person and virtual care. https://www.ontariofamilyphysicians.ca/considerations-for-in-person-visits-aug2021.pdf

### Leading the COVID-19 Vaccine Rollout

In January 2021, the Government of Ontario released guidance to begin immunization in LTC homes (Ontario Ministry of Health, 2021). Leading the vaccine rollout became a priority for NPs, in addition to their existing clinical role in the LTC home. This directive indicated, “immunization of residents is optimally done in the LTCH by trained staff who know the residents, e.g. nurses or physicians” mentioning that “efforts to reduce vaccine hesitancy among staff and essential caregivers may be particularly important to facilitate uptake” (Ontario Ministry of Health, 2021). As primary caregivers and clinical leaders in many LTC homes, the NP role evolved to include conducting and/or supporting the onsite vaccine clinics for residents, staff, and essential caregivers, and minimizing staff vaccine hesitancy.

All NPs described specific responsibilities related to the rollout including actively obtaining consent from staff, residents, and caregivers, administering vaccines, and responding to any adverse reactions. For some NPs, running a vaccine clinic was “another tool in the toolbox” (NP 09), demonstrating their ability to adapt and fill in gaps were required to ultimately improve resident outcomes. Many NPs spoke about the sense of relief that they experienced during the vaccine clinics, as they offered a reprieve from the difficulties of the past year. As one NP stated:

“To be part of [the vaccine rollout], was really special … it helped bring closure to some of the traumas, and the stressful work that we’ve gone through” (NP 04).

One unanticipated consequence of the vaccine strategy and policy implementation was the hesitancy of some staff to be vaccinated. NPs reported on their role in decreasing hesitancy among staff through formal and informal education and remained current with the evolving evidence to provide up-to-date information to staff. NPs described “spending a lot of time searching … for this information to teach people … to try to find all the right information, and make sure it’s all reliable” (NP 13). Many NPs also described being approached by individual staff for reassurance and questions about vaccines; as one NP described, a colleague asked, “Sell me on this vaccine, I’m a little worried about it” (NP 12). Informal education and being onsite was described by NPs as integral in reducing vaccine hesitancy, building trust, and providing staff with an opportunity to make independent and informed decisions about their health, “I think that’s the beauty of being present, you’re just being present and accessible, but you’re asked for advice along the way” (NP 06).

### Promoting Staff Wellbeing During COVID-19 Fatigue

Specific protocols introduced early in January 2021 included screening staff via rapid antigen test once per day, and ensuring proper eye protection, as face shields were donned when providing care for residents with suspected or confirmed COVID-19 infection (Ontario Ministry of Health and Long-Term, 2020a, 2020b). Beyond IPAC protocols established by this point in the pandemic, such as universal masking and physical distancing, these protocols influenced the NPs’ work to focus on ensuring these additional protocols were in place, alongside administrators. NPs often stepped in to help staff experiencing COVID-19 fatigue, which is characterized as tiredness in response to adhering to constantly evolving COVID-19 related protocols and regulations. As one NP stated, “I think people are getting COVID fatigue. They’re tired of getting nasal swabs done, they’re tired of wearing PPE [personal protective equipment]” (NP 11).

The COVID-19 fatigue was further amplified by small-scale outbreaks, which triggered strict testing, cohorting, and isolation precautions (Ontario Ministry of Health and Long-Term, 2020a). To address this, NPs continued to provide IPAC education and reminders to staff. As one NP stated:

“There’s a number of opportunities that staff need to be reminded of what the practice is … I am hearing ‘Oh, we’re tired of this,’ and I said, ‘But the virus isn’t tired.’ There needs to be a lot of reminding and reinforcement” (NP 09).

NPs noted that one year after the first wave, staff were more both physically and emotionally exhausted. NPs reported staff being injured more frequently on the job, as in one home, “they had a nurse collapse and they had to take her out with an ambulance … the nurses are working long or extra shifts” (NP 08). Another NP recounted staffs’ mounting moral distress: “To see someone that you’ve cared for, for years suffer and die, I think [it] took a toll on a lot of PSWs [personal support workers] and nursing staff” (NP 04). As a further outcome of the aggravated levels of staff distress, NPs observed an increased struggle to maintain sufficient staffing levels. It continued to be “really hard to get registered staff” (NP 05). Some NPs noted that many Registered Practical Nurses (RPNs) had decided to leave the LTC homes to work in hospitals and vaccination clinics, often for higher pay and better working conditions, leading to more vacancies in staffing.

NPs responded to the workforce’s moral distress and exhaustion by providing emotional support: “Often it’s just to have an open door and they can come and vent in my office … sometimes they’ll just want to come and sit and not say anything … I’m always there to support them” (NP 03). Although NPs stated they continued to promote capacity building and education, much of their support was informal in nature, “through coaching, debriefing, trying to provide encouragement, information” (NP 09). In addition, NPs stated that staff perceived having them in the building as beneficial because their mere presence was reassuring. As one NP noted, “There’s a bit of a psychological effect having an NP in the building. This helps the staff feel more confident [in dealing with difficult situations], that if something were to happen, they would have someone to assess or provide advice or education … It helps with their morale” (NP 12).

### Complexities Surrounding New Admissions

During the second wave, administrators of LTC homes were directed to expedite the process of admitting hospitalized older adults waiting for a LTC home bed as acute care hospitals were overwhelmed with COVID-19 patients (Ontario Ministry of Health and Long-Term, 2020a). Furthermore, because of the ongoing COVID-19 outbreaks, significant changes in the process of resident admissions were enacted. This included a specific policy requiring all newly admitted residents to undergo a 14-day quarantine period requiring a negative test for COVID-19 upon completion, during which residents were isolated from others in the home as well as their family members (Ontario Ministry of Health and Long-Term, 2020a). This drastic change in environment and lack of socialization put the new residents under considerable stress, and as a consequence, the staff as well. NPs spoke about the worsening of resident wellbeing in various capacities due to their isolation, which was described by some participants as “inhumane.” One NP depicted the challenges associated with the new admissions: “You definitely would see more behavioural and psychological symptoms of dementia … those symptoms worsened for people who didn’t have their usual family member coming in … or who were isolated, they became more agitated” (NP 12). NPs also described residents experiencing increased falls, hypoactivity, and decreased fluid and nutrient intake during the 14 days of isolation.

To address these new challenges, NPs collaborated with staff to implement observation systems, medication review and adjustment, allocation of care aides for high-risk residents, and redeployment of social workers. NPs and staff in the home created a welcoming environment and provided person-centered care. NPs used their expertise to implement assessments, such as the dementia observation system for monitoring behavioral and psychological symptoms in dementia (BPSD), medication review and deprescribing, and supplementary staffing where possible to ensure optimized individual care. However, these resident-centered practices were often faced with challenges during this time due to external policies prioritizing safety over welfare of residents:

“Everything the province has done to date is focused on creating a safe environment and done that at the expense of quality of life. And the reality for our residents is that … quality of life is far more important than safety” (NP 07).

### Negotiating Evolving Collaborative Relationships

In response to a changing care landscape, NPs were required to navigate evolving complex relationships with physician partners in order to continue to provide best resident care. During the second wave of the COVID-19 pandemic, changes in recommendations for primary care providers meant physicians could return to LTC homes to provide care in-person (Ontario Ministry of Health, 2020b). Previous recommendations had promoted a “virtual care first approach” for physicians, however, beginning with the second wave, in-person care was deemed to be increasingly safe, and physicians were expected to return to in-person care where appropriate, dependent on “clinical needs and patient preference” (Ontario College of Family, 2021; Ontario Ministry of Health, 2020b). Continuing to practice to their full scope, NPs adapted to these changes amongst LTC home care providers resulting in “a more of a collaborative effort in looking after the residents” (NP 11).

As many physicians began working virtually throughout the pandemic, their relationship with NPs flourished, resulting in enhanced collaborations, which were not present pre-pandemic for some NPs. Many NPs perceived that they had gained the trust of physicians:

“Prior to the pandemic if someone needed to be seen, a physician would come in. Post-pandemic, if someone needs to be seen, I will see them and assess them and communicate with their physician … they have allowed me to assess and work with them, because I can be the eyes in the facility” (NP 06).

Although physicians began re-entering LTC homes in the second wave, many NPs continued as the primary care provider, as physicians had come to depend on the competence of NPs demonstrated throughout the pandemic: “I found that I was really the primary provider for a lot of these residents … especially during the second [wave]” (NP 02). NPs reported that as a result not all physicians increased their time in the LTC homes to pre-pandemic standards, despite being able to provide direct resident care. As one NP reported, “I think that certainly at a level, they recognize that the home ran without them” (NP 13). However, this increased reliance resulted in an increased workload and a shift in NP role expectations. For instance, one NP stated: “Because physicians have become comfortable with my acute care skills, I do an awful lot of acute care interventions” (NP 07).

With their continually increasing role and responsibilities throughout the second wave, NPs also continued to develop collaborative relationships established with acute care specialists through virtual care to attend to the needs of their residents, provide their own input on devising plans of care, and to avert hospital transfers when possible. NPs depicted this collaborative care process favorably and described specialists, including geriatric psychiatrists and palliative care physicians, as “actually appreciating our input,” particularly in the absence of families and caregivers to advocate on patients’ behalves (NP 06). Further, virtual consults with acute care specialists were described as an invaluable resource throughout the pandemic in making specialized care accessible to all residents: “For people that either have financial issues, or they don’t have families around to bring them to appointments, or things like that, it certainly has been a lifesaver for a lot of my residents. I don’t think I would have been able to consult specialists like that if it wasn’t for e-consult” (NP 03). This enhanced close collaboration not only improved access to care for LTC home residents but also aided acute care providers in better understanding the environment of LTC homes: “And you know their practices have changed with this realization that you can’t just send the resident back in the middle of the night at 2 AM with new prescriptions assuming that it will just get done” (NP 04).

Beyond physician collaboration in clinical care, NPs found that the pandemic offered them the opportunity to share “innovative practice changes and solutions and models of care that were embraced and supported by other collaborative partners,” including acute care and emergency specialists (NP 09). Such initiatives included the development of the Mobile Enhancement and Support Teams (MEST teams) during the second wave, which focused on capacity building in LTC and retirement homes.

Despite this progress in NPs’ relationships with clinical providers, some NPs remained challenged by the lack of recognition of their role within the home and by external funding decisions. NPs voiced their ongoing frustrations about their role as one that remains hidden within the LTC workforce: “There was always a lack of appreciation for what nurses were actually doing. And I think the same thing is happening to NPs” (NP 07). In particular, these challenges have been ongoing in the role of NPs in LTC homes, in that NPs “always sell what we do… you can’t just go to work and be respected, you have to work into that as a role” (NP 06). In order to maintain the newly granted full scope to provide care to residents, NPs were required to advocate for themselves and persevere in the face of these obstacles. As another NP stated:

“Now I’m choosing to speak up … and not waiting for [LTC home management] to ask my opinion, because it’s not happening … so, they’re starting to realize how valuable I can be in the team” (NP 03).

## Discussion

In response to changing directives, policies, and recommendations during the second wave, NPs continued to be flexible and adapt their responsibilities and practices. This study’s results highlight the NPs’ contribution throughout the second wave of the pandemic in improving residents’ quality of life and supporting staff. NPs continued their efforts to contain the spread of the virus and prevent in-house outbreaks; they were available to staff who needed support; they embraced new responsibilities to ensure the safety of new residents as they transitioned into the LTC home environment; and garnered new collaborations with the physicians within and outside the homes.

COVID-19 vaccine hesitancy was common among healthcare professionals despite their vulnerability to infection; one study reported that two in five healthcare professionals intended to delay their COVID-19 vaccine (Parente et al., 2021). NPs led initiatives to discuss how vaccine uptake by staff could decrease the possibility of outbreaks and expanded their role to include working with staff to reduce any hesitancy they experienced. The effectiveness of NPs’ efforts is consistent with recent findings from staff of skilled nursing facilities. In these institutions, which provide transitional medical care and rehabilitation, it was suggested that increasing vaccine confidence could be achieved through partnerships and role modeling of facility and opinion leaders (Harrison et al., 2021). Information provided by healthcare clinicians directly to staff was identified as a critical step in addressing hesitancy (Berry et al., 2021).

The NPs’ support of staff’s emotional wellbeing during the COVID-19 pandemic has far-reaching implications for the long-standing and ever-growing staffing crisis in LTC homes. Even prior to the COVID-19 pandemic, staffing levels and retention were inadequate for the increasing demand for LTC services and increasing resident acuity (Organisation for Economic Co-operation and Development [OECD], 2020). This staffing shortage was clearly exacerbated during the pandemic, in a situation described by the Ontario Long-Term Care COVID-19 Commission as “untenable” (Ontario’s Long-Term Care COVID-19 Commission, 2021). This difficulty in attracting and retaining staff has been clearly linked to burnout, emotional exhaustion, and cynicism (Kelly et al., 2021). However, leadership and culture, amongst other organizational factors, have been demonstrated to be most significant in determining frontline workers’ satisfaction and retention, beyond such individual factors (Chamberlain et al., 2016). This leadership includes qualities inherent to the NP role: empowerment and education of frontline staff, prioritization of emotional and moral support, and the promotion of collaborative environments (Doody & Doody, 2012). As part of the LTC home leadership team, the NP role is ideally placed to indirectly improve staff morale, and ultimately impact staff recruitment and retention.

The findings also highlighted the unique role NPs undertook in admitting new residents into LTC homes and working with staff to reduce the impact of isolation. The usual admission policies in most LTC homes allow family members to enter the facility to help residents in transitioning to their new home (Government of Ontario, 2019b). However, the reported changes in the admissions process led to an increase in social isolation, which can contribute to negative outcomes in older adults living in LTC homes, such as depression, BPSD, and cognitive decline (Chau et al., 2021). Bethell et al. suggested strategies to mitigate the negative effects of isolation, including pain management and addressing communication impairments (2019), both of which were addressed by the NPs through adjustments to care in close collaboration with LTC home teams.

Finally, this study has important implications for the workforce in LTC homes as directives, policies, and recommendations enacted in response to the COVID-19 pandemic provided an opportunity to examine a new model of care, expanding beyond the conventional model employing physicians in the role of Medical Director. Considering only 30% of residents in LTC homes in Ontario have same day access to physicians, a better understanding of alternative models for timely care is imperative (Kobewka et al., 2020). We have provided evidence underscoring the importance of the role of the NP in working collaboratively with physicians in addressing the need for renewed models of care (McGilton et al., 2022)**.** Although many NPs felt a lack of appreciation during this time, the unique capacities of this role cannot be overlooked, nor the success of the NP as MRP. So, while it has been suggested that more physician care will solve the chronic challenges facing the LTC workforce, it is crucial to consider collaborative, interdisciplinary models (Razak et al., 2020). Future NP-physician models of care require trust from the primary physicians to empower NPs’ autonomy and ensure balanced collaboration and respect (Lovink et al., 2019). Since the introduction to the LTC setting, the NP role has been increasingly employed in conjunction with physician practice, indicating increasing collaboration between the roles rather than role substitution; our results confirm this trend (Intrator et al., 2015). One successful collaborative model is the Evercare model, now known as Optum CarePlus, which implements a NP-geriatrician collaborative team to supplement existing primary care in LTC homes, and to provide additional comprehensive and preventative care (Polich et al., 1990). More recently, the Co-management model has proposed a similar collaboration between physicians and NPs (Norful et al., 2018). The emphasis on the NP role in these models is effective in enhancing quality of care and improved resident outcomes; Evercare reduced resident hospitalizations by 40% and ED visits by 50% or greater (Kane et al., 2003). The NP Co-management model has shown qualitative improvement in primary care for common geriatric conditions such as falls, UTIs and dementia (Reuben et al., 2013). Future research is required to evaluate these models in LTC homes, as there is most likely not a one size fits all model. What appears important is that NPs are empowered to work at their full scope of practice in the LTC sector; future NP-physician models of care require trust from the primary physicians to empower NPs’ autonomy and ensure balanced collaboration (Lovink et al., 2019). Our findings support the inclusion of NPs as a mandatory member of the LTC home workforce and remuneration on par to NPs in the acute care sector (Ontario Ministry of Health and Long-Term, 2020c).

### Strengths and Limitations

This study recruited NPs within one province in Canada, so the results may not be transferable. However, NPs were recruited from both urban and rural jurisdictions throughout the province. Further, NPs are employed in several countries and practiced in LTC homes throughout the pandemic, so we anticipate our findings are generalizable to other countries. Although our sample size was moderate in context of the Ontario NP population, our interviews were fruitful and reaching saturation increased credibility of our findings. We did not interview residents or other members of the LTC home teams, which may have led to a more objective description of the NP role and would have validated the perspectives shared by the NPs.

## Conclusions

The unique role of the NP must be considered when developing workforce solutions to LTC homes’ longstanding issues illuminated throughout the COVID-19 pandemic. The results of this study demonstrated that NPs adapted and expanded their practices to maintain the quality of residents’ health care, supported staff and management, and negotiated collaborative relationships with care providers. Future models of care warrant further development and a global investment to include NPs as integral clinicians embedded within the LTC settings with the ability to work to their full scope of practice.

## Supplemental Material

Supplemental Material—Nurse Practitioners Navigating the Consequences of Directives, Policies, and Recommendations Related to the COVID-19 Pandemic in Long-Term Care Homes Click here for additional data file. Supplemental Material for Nurse Practitioners Navigating the Consequences of Directives, Policies, and Recommendations Related to the COVID-19 Pandemic in Long-Term Care Homes by Katherine S. McGilton, Alexandra Krassikova, Aria Wills, Vanessa Durante, Lydia Yeung, Shirin Vellani, Souraya Sidani, and Astrid Escrig-Pinol in Journal of Applied Gerontology
