# Supplementary material for: Nurse Practitioners Navigating the Consequences of Directives, Policies, and Recommendations Related to the COVID-19 Pandemic in Long-Term Care Homes
Source: J Appl Gerontol. 2022 Jun 25;41(11):2296–306. doi: 10.1177/07334648221110210 (PMC9234378; doi:10.1177/07334648221110210)
Supplement: Supplemental Material—Nurse Practitioners Navigating the Consequences of Directives, Policies, and Recommendations Related to the COVID-19 Pandemic in Long-Term Care Homes [file sj-pdf-1-jag-10.1177_07334648221110210.pdf]

Supplementary Appendix 1. **Semi-structured Interview guide**

1. Has your role in the long-term care (LTC) home(s) changed since last time we spoke (August – September 2020)?

(Probe: number of homes working in, number of hours working, virtual and/or on-site care?)

2. Which level of pandemic preparedness did you experience while working in the LTC home(s)? Please describe.

(Probe: management preparedness, policies and procedures, training for staff, staff capacity)

3. Have you observed the home(s) implement specific changes to optimize the care provided to residents?

(Probe: number of full-time staff, increase physician presence, increased family presence)

4. Which policies, procedures, or protocols were established in response to the pandemic in the home(s) that you work at? Please describe.

(Probes: changes in relation to coroner's office; changes in relation to ministry inspectors)

5. What is your role in vaccine rollout at your home(s)?

(Probe: securing supply, storage, administration)

6. What is your experience in relation to staff and residents' buy in towards the vaccine?

7. Did you play a role in addressing staff and residents' concerns?

(Probe: vaccine safety, availability)

8. Did you have responsibilities supporting staff during the second wave?

(Probe: identifying and addressing moral distress, compassion fatigue, NP experiencing distress)

9. Did you observe the home(s) implementing any interventions to support staff?

10. Did your role change in supporting quality of care for the residents during the second wave of COVID-19?

(Probe: new initiatives in optimizing resident care; new admission processes, access to specialists and/or physicians)

11. What changes, positive and/or negative, did you encounter working as an NP in LTCH(s) during the second wave of pandemic?

12. Which changes in LTCHs policies and regulations would you like to see happening in the future? Why?
